# Influence of body weight on lung mechanics and respiratory function in ARDS patients

**DOI:** 10.1186/cc14319

**Published:** 2015-03-16

**Authors:** I Algieri, D Chiumello, C Mietto, E Carlesso, A Colombo, G Babini, M Cressoni

**Affiliations:** 1 Università degli Studi di Milano, Milan, Italy; 2 Fondazione IRCCS, 'Ospedale Maggiore Policlinico Mangiagalli Regina Elena' di Milano, Milan, Italy

## Introduction

Traditionally, ARDS obese patients are ventilated with higher tidal volumes and higher PEEP due to expected increased in pleural pressure. However, data from the literature regarding the influence of body mass on lung mechanics and, particularly, on chest wall elastance are not univocal [1,2]. Failure to account for how the increase in body weight could affect the respiratory function can result in injurious mechanical ventilation and in the onset of VILI. The aim of this study was to evaluate the role of the body weight on respiratory mechanics in ARDS patients.

## Methods

A group of deeply sedated and paralyzed ARDS patients was divided into three classes according to the body mass index: normal weight (between 18.5 and 24.9 kg/m^2^), overweight (between 25.0 and 29.9 kg/m^2^) and obese (between 30.0 and 39.9 kg/m^2^). They were mechanically ventilated in volume-controlled mode with a tidal volume of 6 to 8 ml/kg according to predicted body weight. Respiratory mechanics and gas exchange were assessed at PEEP levels of 5 and 15 cmH_2_O.

## Results

A total of 101 ARDS patients was enrolled; 44 (43.6%) patients were normal weight, 36 (35.6%) overweight and 21 (20.8%) obese. Lung and chest wall elastance were not different between groups, both at PEEP levels of 5 cmH_2_O and 15 cmH_2_O (*P *= 0.580 and *P *= 0.113, respectively), and the end-inspiratory transpulmonary pressure was also similar. We did not observe any difference between groups regarding PaO_2_/FiO_2_ ratio (*P *= 0.178 at PEEP 5 cmH_2_O; *P *= 0.073 at PEEP 15 cmH_2_O) and PaCO_2_ (*P *= 0.491 at PEEP 5 cmH_2_O; *P *= 0.237 at PEEP 15 cmH_2_O).

## Conclusion

In ARDS obese patients the chest wall elastance and the end-inspiratory transpulmonary pressure are not affected by the body weight, suggesting that normal weight and obese patients present similar risks for lung stress and VILI onset. The severity of hypoxemia was not different between groups.
